# Function of BMP4 in the Formation of Vasculogenic Mimicry in Hepatocellular Carcinoma

**DOI:** 10.7150/jca.40558

**Published:** 2020-02-10

**Authors:** Xiao Li, Baocun Sun, Xiulan Zhao, Jindan An, Yanhui Zhang, Qiang Gu, Nan Zhao, Yong Wang, Fang Liu

**Affiliations:** 1 Department Of Pathology, General Hospital Of Tianjin Medical University, Tianjin, 300052, China; 2 Department Of Pathology, Tianjin Medical University, Tianjin, 300070, China; 3 Department of Pathology, Cancer Hospital of Tianjin Medical University, Tianjin, 300060, China

**Keywords:** Bone morphogenetic protein 4, hepatocellular carcinoma, vasculogenic mimicry, epithelial-mesenchymal transition, stemness

## Abstract

Vasculogenic mimicry (VM) is linked to vascular invasion of human hepatocellular carcinoma (HCC). BMP4, one BMP family member, is upregulated in several cancers. The purpose of this report is to identify the function of BMP4 in the formation of VM in HCC and the mechanism underling this regulation. In our report, BMP4 up-regulation resulted in an increase in migration, invasion and channel-like structure formation as well as induced epithelial-mesenchymal transition (EMT) process and stem cell-associated proteins OCT4 and SOX2 expression in HCC cells. In addition, The VM-associated proteins, including EphA2, VE-cadherin and MMP2, also could be effectively enhanced by the overexpression of BMP4. Furthermore, according to the TCGA database, higher expression of BMP4 is seen in HCC in contrast to normal liver samples. Immunohistochemistry revealed that BMP4 was positively associated with VM formation, age, histological differentiation, HCC stage, and shorter survival duration. These data demonstrated that BMP4 could promote VM network formation in HCC through induction of stemness in EMT and modulating the EphA2/VE-cadherin/MMP2 signaling pathway.

## Introduction

Hepatocellular carcinoma (HCC) has a high incidence worldwide 1, 2. It is frequently linked to vascular invasion and high metastasis 3. Previous studies of our team have demonstrated that vasculogenic mimicry (VM) has a significant impact on HCC blood supply and is essential for HCC cell survival 4. VM, unlike traditional angiogenesis, describes the formation of blood vessels by highly invasive tumor cells 5. Patients who display VM have more invasive cancer phenotypes 6. A number of VM-associated proteins have been identified, including EphA2, VE-cadherin, MMP2, OCT4 and SOX2 7-9. Studies have also demonstrated that VM in tumor is linked to epithelial-mesenchymal transition (EMT) and stem cell-like properties 10, 11. A signaling pathway of the VM was first proposed by Hendrix and colleagues 12, which suggested that VE-cadherin and EphA2 colocalize and subsequently lead to activation of matrix metalloproteinase 2 (MMP2) 13. Nevertheless, the exact underlining mechanism in VM formation in HCC requires further analysis.

Bone morphogenetic proteins (BMPs) are part of the transforming growth factor β (TGF-β) superfamily. Upon binding to the associated membrane receptor, BMPs phosphorylate and activate Smad1/5/8 protein complex, which assemble with SMAD4 and activate or suppress downstream targets 14, 15. BMPs have significant effects on cell proliferation, differentiation and embryonic development 14. In addition, researchers have claimed that BMPs also play a variety of roles in carcinomas 16-21. One BMP family member, BMP4, is essential for the morphogenesis of hepatic diverticulum, also known as a liver bud 22-24. Studies have indicated that BMP4 enhances invasion and metastasis 25-29. In particular, BMP4 serves as an angiogenic factor that promotes vascular network formation in HCC 26, 30. More interestingly, BMP4 is critical in epithelial-mesenchymal transition (EMT) process, which possesses stem-cell like characteristics 31-35. Moreover, EMT and stem-like features are both involved in VM 10, 11. Research found that BMP4 is linked to VM channel formation, which appears to be mediated through increasing VE-cadherin and EphA2 expressions 25. Another study also supported that BMP4 increases MMP2 expression 36, which is involved in VM and the downstream EphA2/ VE-cadherin/ MMP2 pathway 12. Based on the above data, it sparked our interest in exploring whether BMP4 could promote VM formation in HCC via inducing EMT stemness and modulating the EphA2/ VE-cadherin/ MMP2 pathway. In this report, we investigated the relationship between BMP4 and VM in HCC tumors and examined the effect of BMP4 on migration, invasion and VM formation in human HCC cell lines. Finally, we evaluated whether BMP4 could promote the formation of VM through induction of EMT stemness and modulating the EphA2/ VE-cadherin/ MMP2 pathway.

## Methods

### Patient tissues

Patient tissues were collected from 92 HCC patients receiving live resection in Tianjin Cancer Hospital and Tianjin General Hospital. We gathered exhaustive pathologic and clinical data of the 92 patients. These patients were never treated with antitumor therapy before the surgery. The protocol was approved by the Ethics Committee of Tianjin Cancer Hospital and Tianjin General Hospital.

### Immunohistochemical (IHC) staining

Paraffin-embedded tumor samples (4-μm) were deparaffinized, hydrated and treated with endogenous peroxidases. Then, the slides were subject to antigen retrieval. Next, the slides were subject to 10% normal goat serum to inactivate endogenous peroxidase and treated overnight with rabbit monoclonal against BMP4 antibody (ab124715, Abcam, USA) at 4°C. Following incubation, the corresponding secondary antibody was added. Then, we used 3,3′-diaminobenzidine chromogen to detect peroxidase activity. Ultimately, slides were counterstained with hematoxylin. Staining result was assessed based on a previously described method 37.

### CD31/ Periodic Acid-Schiff (PAS) double staining and definition of VM

IHC staining of CD31 (ZM-0014, Zhongshan Chemical Co., China) was applied as described above and slides were then subject to PAS, ultimately hematoxylin staining was performed.

VM is characterized based on previously established definition 38. Briefly, VM is known to form channels under the tumor cell microenvironment in exhibiting red blood cells. VM is present in CD31/PAS double-stained sections but not CD31-stained sections.

### Cell lines

HepG2 HCC cell line (American Type Culture Collection) and SMMC7721 HCC cell line (Zhongshan Hospital Affiliated to Fudan University, Shanghai, China) were passaged in DMEM (Neuronbc, China) supplemented with 10% fetal bovine serum (FBS) (Gibco, USA). Experiments were performed when cells were 70-80% confluent.

### Expression plasmids and cell transfection

The expression plasmids containing BMP4 cDNA and BMP4 shRNA were obtained from GeneCopoeia, Inc. (catalog no. EX-A0242-M98; HSH053910-CU6). HepG2 cells were transfected with the BMP4 cDNA-containing plasmid to establish the HepG2-BMP4 up-regulation model; SMMC7721 cells were transfected with the BMP4 shRNA-containing plasmid to establish the SMMC7721-shBMP4 down-regulation model.

### Wound healing assay

HepG2, HepG2-BMP4, SMMC7721, and SMMC7721-shBMP4 cells were cultured in six-well culture plates for 24h. Then, a plastic tip was used to scratch a straight line in each well. The speed of wound closure was assessed after 24h and 48 h.

### Invasion assay

The invasion assay was applied in 24-well chambers with 8μm pores (BD Biosciences, USA). First, the top chambers were coated with Matrigel (BD Biosciences, USA). Then, 1 × 10^5^ cells were subject to serum-free DMEM in the top chambers, while DMEM with FBS were subject to bottom chambers. After 48h incubation, invading cells that added to the bottom were fixed and then subject to crystal violet staining. Finally, invading cells were counted and photographed (Nikon, Japanese).

### Three-dimensional (3D) cultures

96-well plates were coated with Matrigel (35μl per well) for 30min at 37°C. Then, HepG2, HepG2-BMP4, SMMC7721, and SMMC7721-shBMP4 cells in DMEM supplemented with 10% FBS were seeded on the surface of solid gel for 24h at 37°C. Finally, the tube-like networks were assessed and photographed (Nikon, Japanese).

### Zymography assays

Serum-free culture medium were clumped and subjected to sodium dodecyl sulfate polyacrylamide gel electrophoresis (SDS-PAGE). Gels were washed in 2.5% Triton X-100 then cultured with developing buffer overnight at 37°C. Subsequently, the Coomassie Brilliant Blue R250 staining was performed. Finally, the gels were washed with water. The proteolytic activity of MMP2 was detected as clear bands on the gel.

### Western blot

HepG2, HepG2-BMP4, SMMC7721, and SMMC7721-shBMP4 cells were lysed in 10% SDS. Cell lysates were first subject to SDS-PAGE, transferred to polyvinylidene fluoride membranes (Millipore, USA) and then blocked in non-fat milk. Primary antibodies including BMP4 (ab124715, Abcam, USA), E-cadherin (zs-78700, Zhongshan Chemical Co., China), Vimentin (2707-1, Epitomics, USA), EphA2 (sc-924, Santa Cruz Biotechnology, USA), VE-cadherin (ab33168, Abcam, USA), MMP2 (sc-13595, Santa Cruz Biotechnology, USA), OCT4 (sc-8629, Santa Cruz Biotechnology, USA) and SOX2 (GTX101507, GeneTex, USA) were added to the membranes at 4°C overnight. The next day, the secondary antibodies including goat anti-rabbit IgG-HRP (ZB-2301, Santa Cruz Biotechnology, USA), goat anti-mouse IgG-HRP (ZB-2305, Zhongshan Chemical Co., China) and rabbit anti-goat IgG-HRP (ZB-2306, Zhongshan Chemical Co., China) were added for 2 h at 37°C. The protein levels were assessed using the enhanced chemiluminescence method.

### Immunofluorescence staining

Cells were seeded on coverslips, permeabilized with ice-cold methanol, and incubated with normal goat serum (5%). The primary antibodies were maintained with the slips overnight at 4°C. The next day, secondary antibodies were added for 2h at 37°C. After immunolabeling, 4′,6-diamidino-2-phenylindole (DAPI; Zhongshan Chemical Co., China) was used for nuclei staining. Finally, the cells were mounted and photographed (Nikon, Japanese).

### Bioinformatics analysis

TCGA (https://cancergenome.nih.gov/) database was performed to predict the expression levels of BMP4 in HCC and normal tissues. A protein-protein interaction (PPI) network of BMP4 correlated genes was constructed according to the STRING (https://string-db.org/) database.

### Statistical analysis

All experiments applied at least in triplicate independently, results were expressed as mean ± standard deviation (SD) and assessed using SPSS17.0 software. Two-sided Pearson Chi-square test and Student's t-test were performed to compare the outcomes. P < 0.05 was regarded as statistically significant.

## Results

### BMP4 was overexpressed in human HCC samples and associated with VM and clinicopathological features

**Fig 1:**
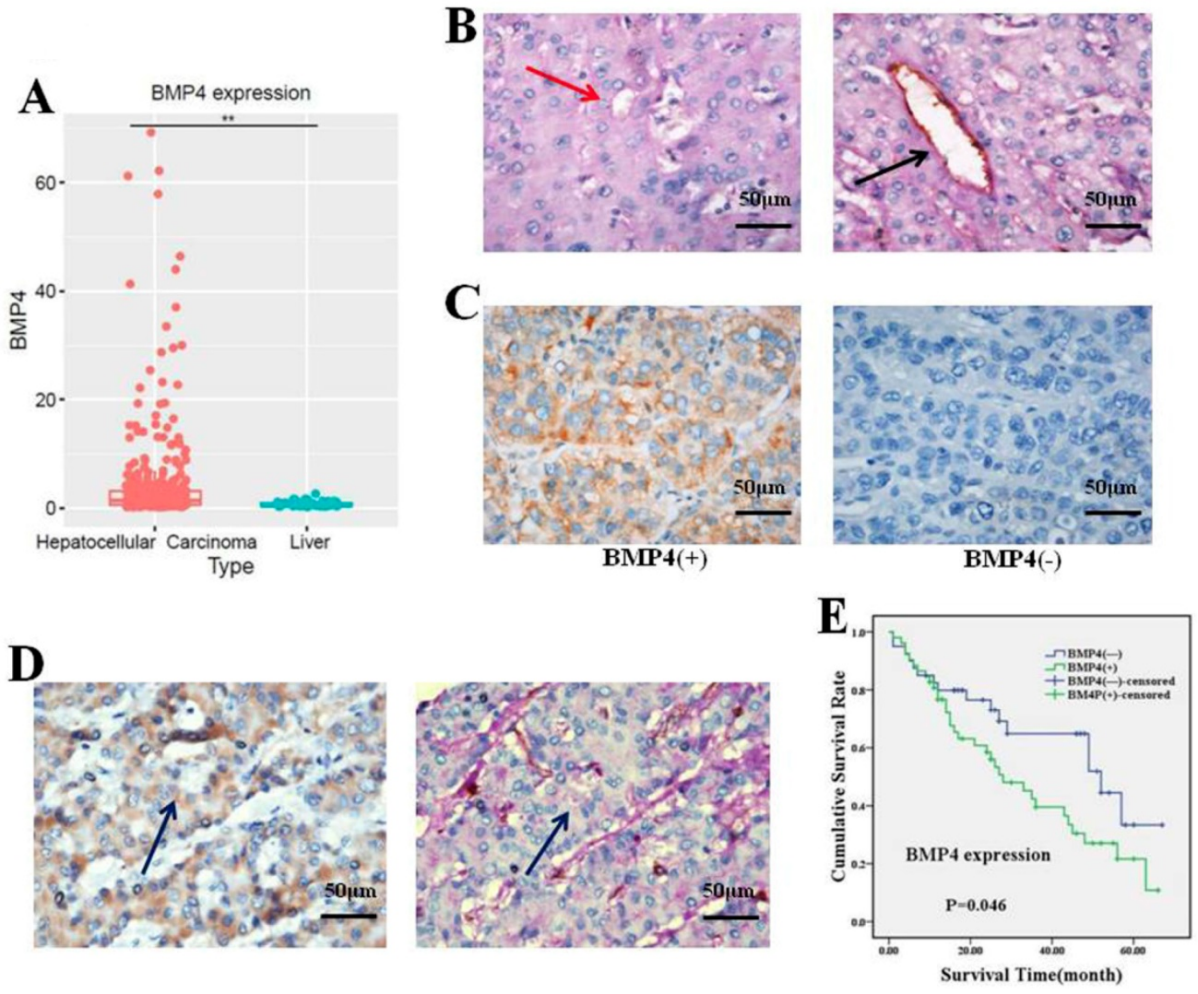
(A) BMP4 was dramatically enhanced in HCC (n=374) in contrast to normal liver samples (n=50) according to the TCGA database. *P<0.05. (B) Evidence of VM (red arrow) and angiogenesis (black arrow) in HCC samples (400x, scale bar=50μm). (C) BMP4 expression in HCC (400x, scale bar=50μm). BMP4 in VM-positive (left) and VM-negative (right) HCC samples. (D) Colocalization of BMP4 and VM formation was identified by IHC in consecutive sections (blue arrow, 400x, scale bar=50μm). (E) BMP4-positive patients had poorer survival compared to BMP4-negative patients according to Kaplan-Meier survival analysis (P=0.046).

To validate the effect of BMP4 on HCC, we investigated BMP4 expression in HCC and normal liver samples by using the TCGA database. We observed that BMP4 was notably enhanced in HCC (p<0.05, Fig. 1A). Subsequently, we evaluated BMP4 expression in HCC using immunohistochemistry analysis. Studies present VM identification in HCC tissue using CD31-PAS double-staining 10, 39. In 17 of 92 (18.48%) HCC samples, VM vessels were observed to be lined by tumor cells without inflammatory cell infiltration or necrosis around the channels (Fig. 1B). Positive expression of BMP4 was displayed in the cytoplasm of HCC cells. According to the existence of BMP4, 92 samples were categorized into two teams: BMP4 positive staining team (n = 52) and BMP4 negative staining team (n = 40) (Fig. 1C). Among the 52 BMP4-positive group, 14 (26.92%, 14/52) showed presence of VM; and among 40 BMP4-negative group, 3 (7.5%, 3/40) showed presence of VM. The outcome indicated that BMP4 was positively associated with VM (P=0.017, Table 1). The colocalization of BMP4 expression and VM was identified by IHC in consecutive sections (Fig. 1D). Next, the correlation between BMP4 and pathological features were analyzed. Based on our findings, BMP4 was significantly correlated with age, histological differentiation, and cancer stage (P = 0.004, 0.044, and 0.019, respectively, Table 2). In patients over 45 years old, BMP4 was much higher than in samples from patients aged 45 years or younger (65.67%, 44/67 versus 32.00%, 8/25, respectively). Among the four HCC histological differentiation and stage types, BMP4 was found at higher expression in III/IV than I/II types (63.93%, 39/61 versus 41.94%, 13/31; 66.67%, 36/54 versus 42.11%, 16/38, respectively). However, the presence of BMP4 did not show any association with other pathological characteristics, such as gender, tumor size, or metastasis (P > 0.05, Table 1). To determine the prognostic role of BMP4, the Kaplan-Meier analysis was applied. Analysis showed that patients of positive BMP4 expression had poorer prognosis than patients of negative BMP4 expression (P = 0.046, Fig. 1E).

### BMP4 up-regulation in HepG2 cells and down-regulation in SMMC7721 cells

**Fig 2:**
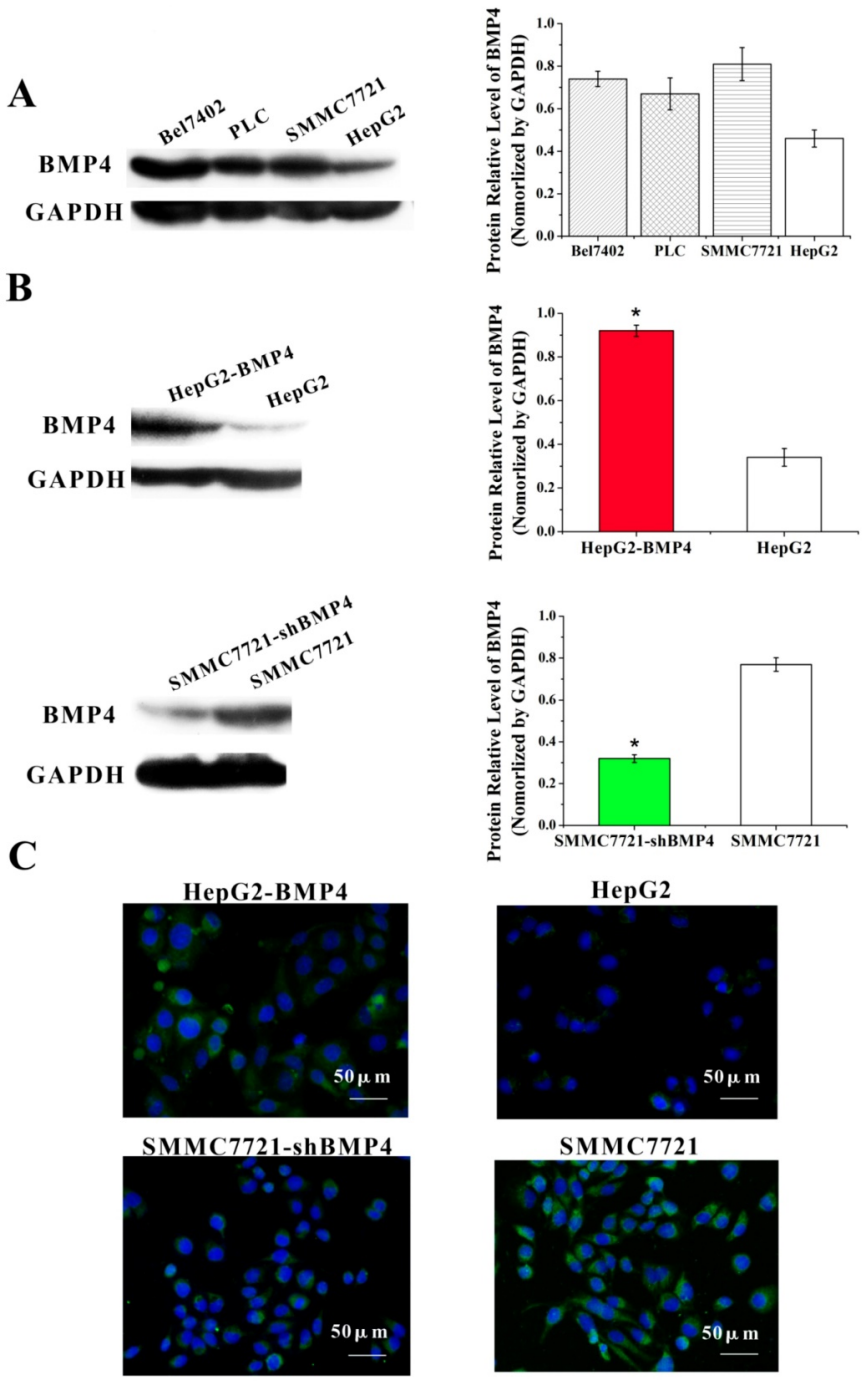
(A) The expression of BMP4 in four HCC cell types was evaluated according to western blot analysis. (B, C) BMP4 protein level was up-regulated in BMP4-overexpressing HepG2 cells; the up-regulation efficiency was validated through western blot and immunofluorescence. BMP4 was knocked down in SMMC7721 cells using shRNA interference; the down-regulation efficiency was also validated through western blot and immunofluorescence. Green: target protein; blue: DAPI-stained nuclear DNA. *P<0.05, scale bar=50μm.

First, we compared BMP4 protein expression in four HCC cell types. Based on western blot, we found that well-differentiated HepG2 cells had lower BMP4 expression compared to poorly differentiated SMMC7721 cells (Fig. 2A). After separated transfection of BMP4 cDNA in HepG2 cells and BMP4 shRNA in SMMC7721 cells, western blot (Figure. 2B) and immunofluorescence (Figure. 2C) confirmed that BMP4 expression in HepG2-BMP4 group was enhanced and BMP4 expression in SMMC7721-shBMP4 group was decreased compared to their respective controls.

### Up-regulation of BMP4 enhanced HCC cells migration and invasion capabilities, while BMP4 down-regulation inhibited migration and invasion capabilities

**Fig 3:**
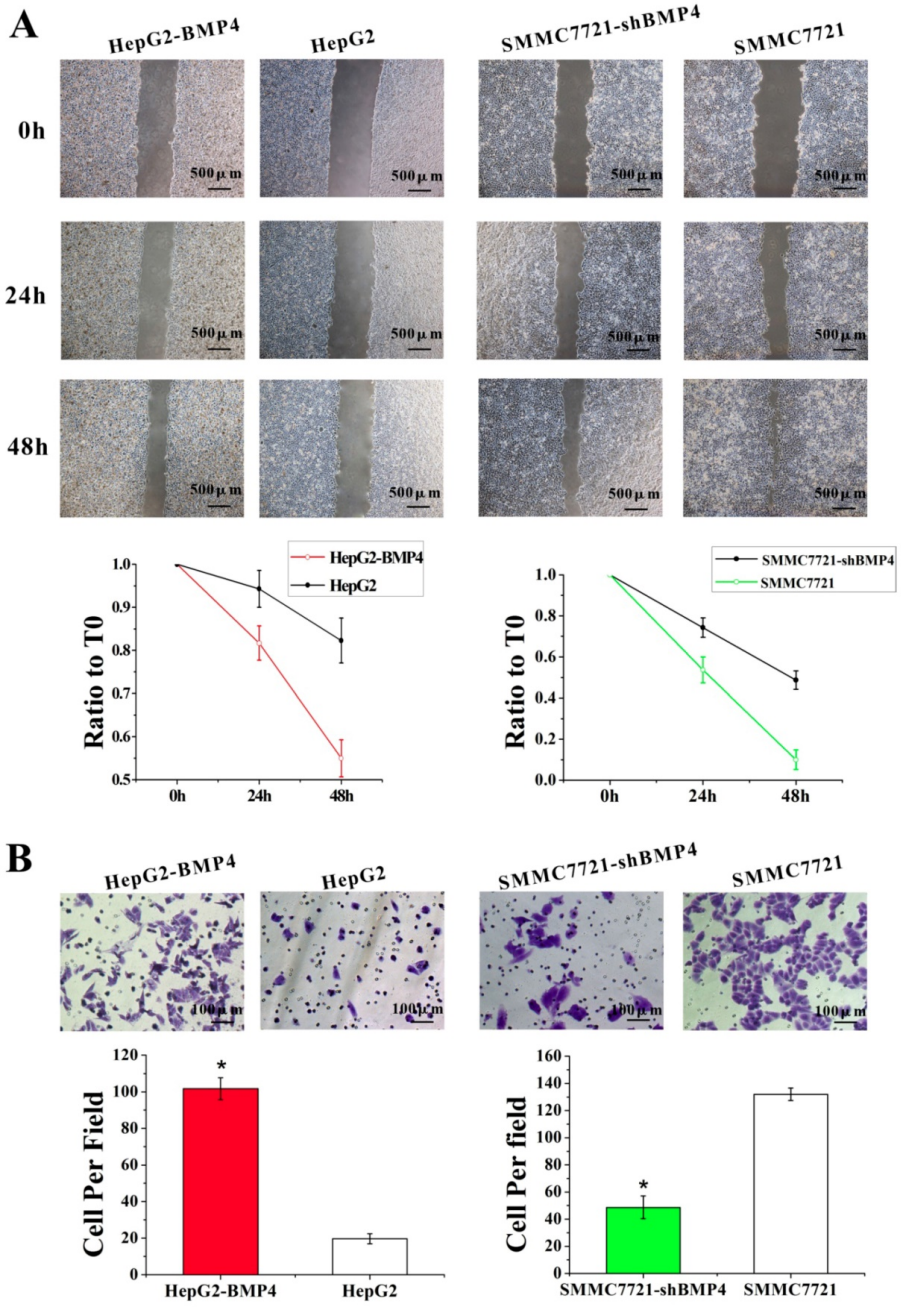
The effects of BMP4 overexpression and knockdown in HCC cell migration and invasion. (A) The wound healing assay demonstrated that in contrast to controls, BMP4 up-regulation resulted in faster wound healing speed and BMP4 knockdown resulted in slower wound healing speed, scale bar=500μm. (B) In the invasion assay, BMP4 overexpression presented a greater increase in cell invasion compared with control HepG2 cells, and BMP4 knockdown presented a notable decrease in contrast to control SMMC7721 cells. *P<0.05, scale bar=100μm.

The function of BMP4 in HCC cell migration and invasion were evaluated by the up-regulation and down-regulation cell culture systems described above. Wound healing assay was utilized for assessing cell migration. Compared with controls, BMP4 up-regulation resulted in faster wound healing while BMP4 down-regulation showed the opposite (Fig. 3A). These results support that BMP4 plays a positive role in cell migration. Similarly, following the invasion assay (Fig. 3B), invading cells were significantly enhanced in HepG2-BMP4 cells but reduced in SMMC7721-shBMP4 cells compared to respective controls. In conclusion, BMP4 up-regulation enhanced HCC cell migration and invasion capabilities, while BMP4 down-regulation inhibited HCC cell migration and invasion capabilities.

### Up-regulation of BMP4 induced VM formation and BMP4 down-regulation impaired VM formation

**Fig 4:**
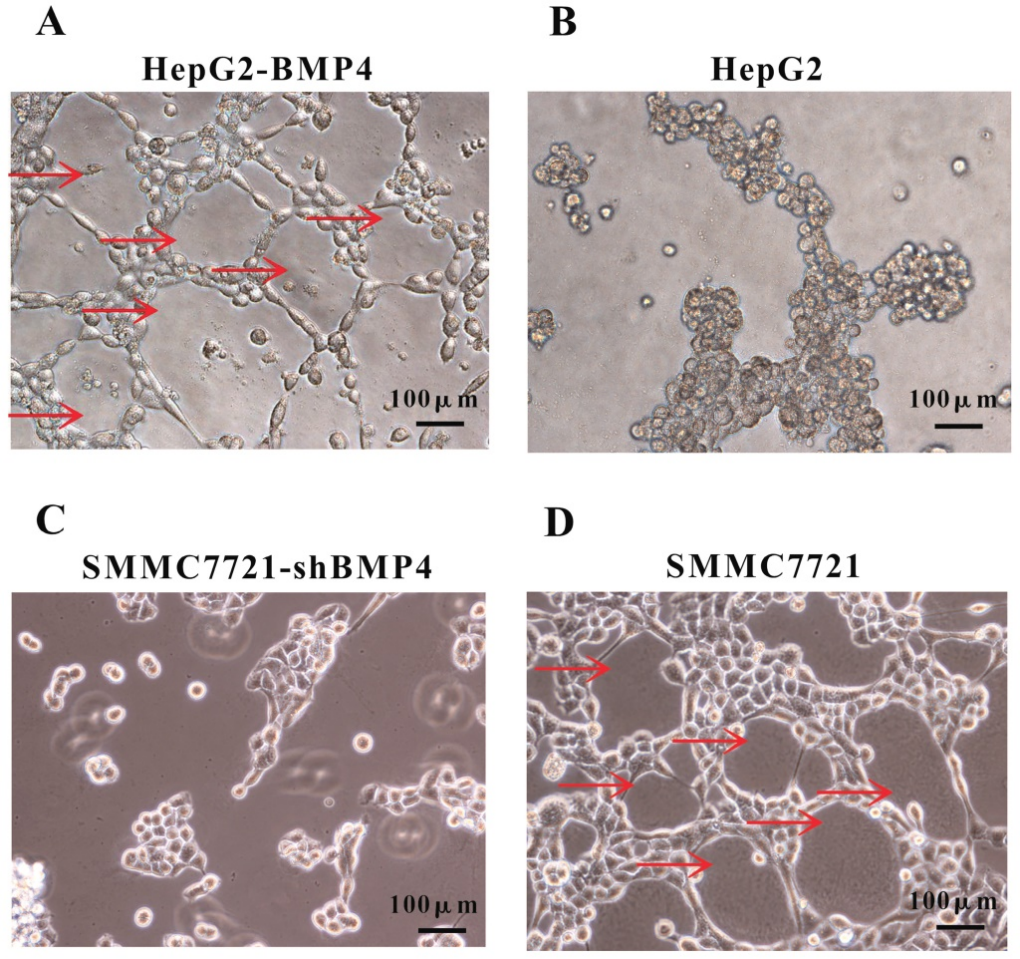
VM formation in 3D cell culture. (A, B) The condition of VM structures in HepG2 up-regulation group and control group. (C, D) The condition of VM structures in SMMC7721 down-regulation group and control group. Red arrows pointed to the VM structures on Matrigel, scale bar=100μm.

A well-established 3D cell culture model was utilized for validating the effect of BMP4 expression on VM formation. In 3D Matrigel cultures, HepG2 cells failed to construct channel-like structures (Fig. 4B), but SMMC7721 cells were able to form such networks (Fig. 4D). We suspected that the lack of vascular network formation in HepG2 cells were due to low BMP4 expression. As expected, overexpression of BMP4 in HepG2 cells resulted in strong VM formation (Fig. 4A). Meanwhile, BMP4 down-regulation in SMMC7721 cells showed a dramatical suppression in pipe-like network formation (Fig. 4C). These data further indicated that BMP4 is an inducing factor of VM network formation in HCC cells.

### Up-regulation of BMP4 induced EMT and increased the expression of stem cell-associated proteins OCT4 and SOX2; down-regulation of BMP4 reversed EMT and decreased the expression of stem cell-associated proteins OCT4 and SOX2

**Fig 5:**
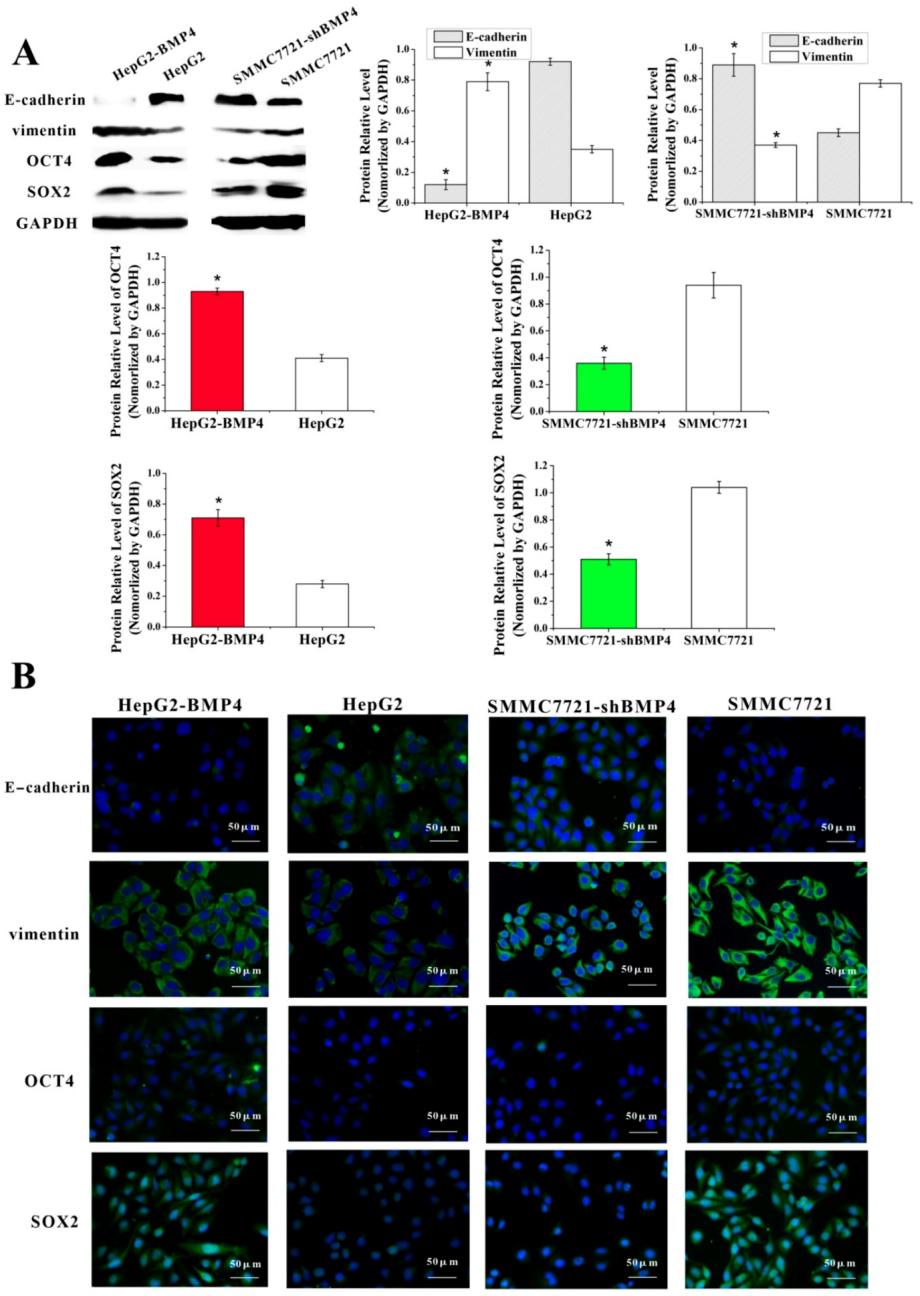
Expression of E-cadherin, vimentin, OCT4 and SOX2 were evaluated according to western blot (A) and immunofluorescence (B). Cells overexpressing BMP4 had enhanced vimentin, OCT4 and SOX2 expression, but decreased E-cadherin expression compared with the controls; cells under-expressing BMP4 had decreased vimentin, OCT4 and SOX2 expression, but increased E-cadherin in contrast to the controls. *P<0.05, scale bar=50μm.

EMT is characterized by losing epithelial properties and taking on an invasive phenotype typical of mesenchymal cells 40. EMT is previously thought to be associated with VM in HCC 10. To validate the effect of BMP4 on EMT process, we examined changes in EMT-associated factors in HepG2-BMP4 and SMMC7721-shBMP4 cells (Fig. 5A, Fig. 5B). Results from HepG2-BMP4 cells indicated that there was a remarkable increase of vimentin and a great decrease of E-cadherin compared to HepG2 control cells. The opposing effect was observed in SMMC7721-shBMP4 cells. Taken together, we hypothesized that overexpression of BMP4 may induce EMT, while BMP4 knockdown may reverse EMT.

Cancer cells undergoing EMT display stem cell-properties including tumor- initiating and self-renewal 41, 42. Thus, we examined stem cell regulators, OCT4 and SOX2, after BMP4 overexpression and knockdown. As expected, analysis revealed that up-regulation of BMP4 increased OCT4 and SOX2. Conversely, knockdown of BMP4 decreased OCT4 and SOX2 expression (Fig. 5A, Fig. 5B). Our results demonstrated that BMP4 may confer stem cell-like behaviors in HCC cells.

### BMP4 may lead to the formation of VM via the EphA2/VE-cadherin/MMP2 pathway

**Fig 6:**
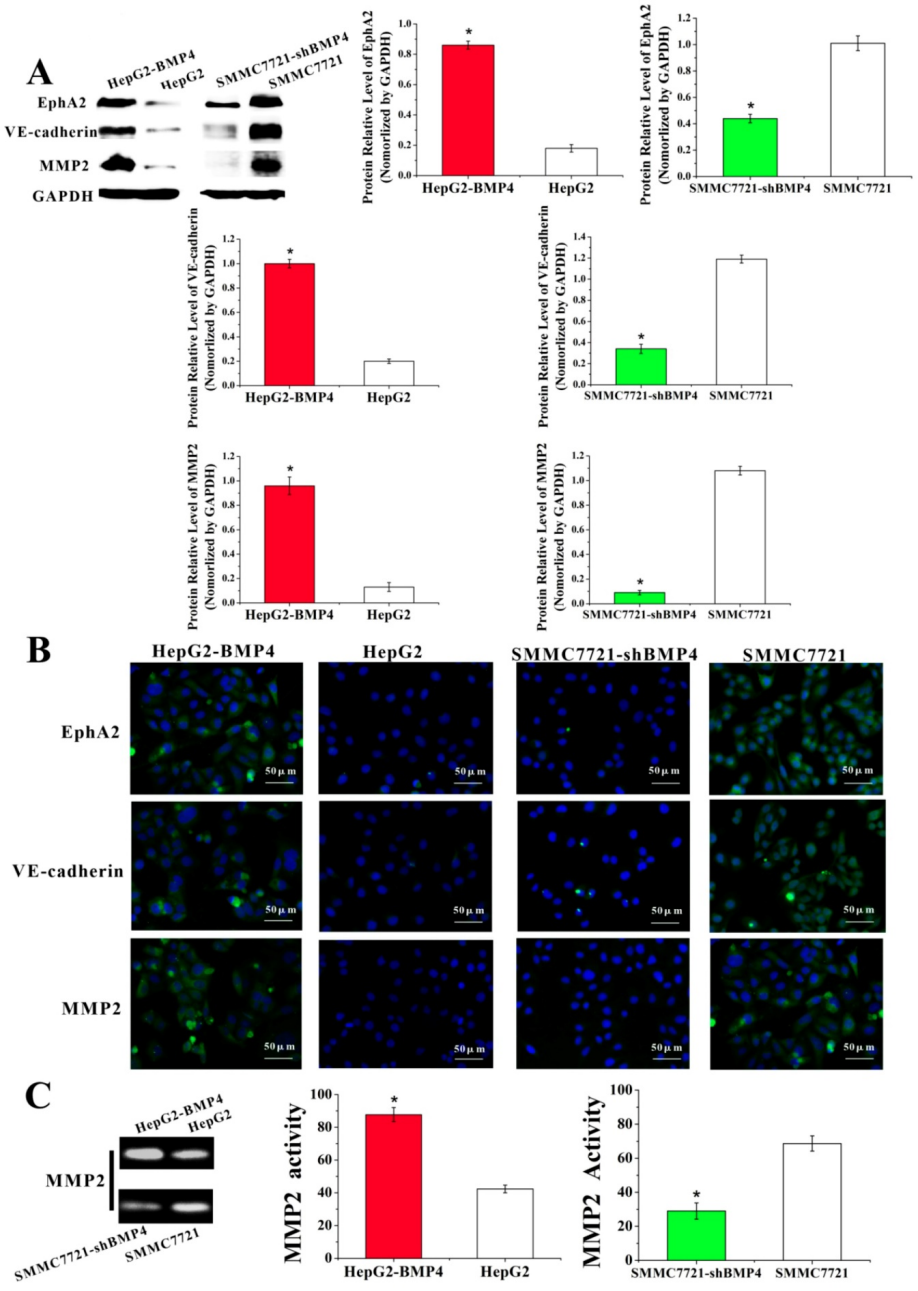
Expression of EphA2, VE-cadherin and MMP2 were evaluated through western blot (A) and immunofluorescence (B). Cells overexpressing BMP4 had higher EphA2, VE-cadherin and MMP2 protein levels in contrast to controls; cells under-expressing BMP4 had lower EphA2, VE-cadherin and MMP2 protein levels compared with controls, scale bar=50μm. (C) MMP2 activity was analyzed by zymography assay. Stronger MMP2 bands were observed after overexpressing BMP4 in HepG2 cells; weaker MMP2 bands were observed after knocking down BMP4 in SMMC7721 cells. *P<0.05.

Currently proposed signaling cascade for the formation of VM involves both EphA2 and VE-cadherin, which induce the expression and activation of MMP2 that ultimately result in VM formation 12. EphA2 is a transmembrane receptor, which regulates cellular migration and invasion. Suppression of EphA2 expression impairs the formation of VM *in vitro* 43-45. In our experiments, EphA2 was dramatically increased in HepG2-BMP4 cells, and the opposite result was found in SMMC7721-shBMP4 cells (Fig. 6A, Fig. 6B).

VE-cadherin is critical in VM formation 46. Remarkably, in HepG2-BMP4 group, VE-cadherin expression was effectively enhanced; while in SMMC7721-shBMP4 group, there was a down-regulation in VE-cadherin compared with control cells (Fig. 6A, Fig. 6B).

MMP2 is a key player in VM formation that acts as an effector molecule 12, 13, 47. As shown in Fig. 6A and Fig. 6B, MMP2 expression was strikingly enhanced after the up-regulation of BMP4 in HepG2 cells. The opposite result was found in SMMC7721-shBMP4 cells due to the down-regulation of BMP4. Furthermore, zymographic assay (Fig. 6C) results were in accordance with western blot and immunofluorescence. Based on the above data, HCC cells may enhance EphA2, VE-cadherin expression, and activate MMP2 via increasing BMP4 expression, which is ultimately promoting the formation of VM.

### Protein-protein interaction (PPI) network analysis

**Fig 7:**
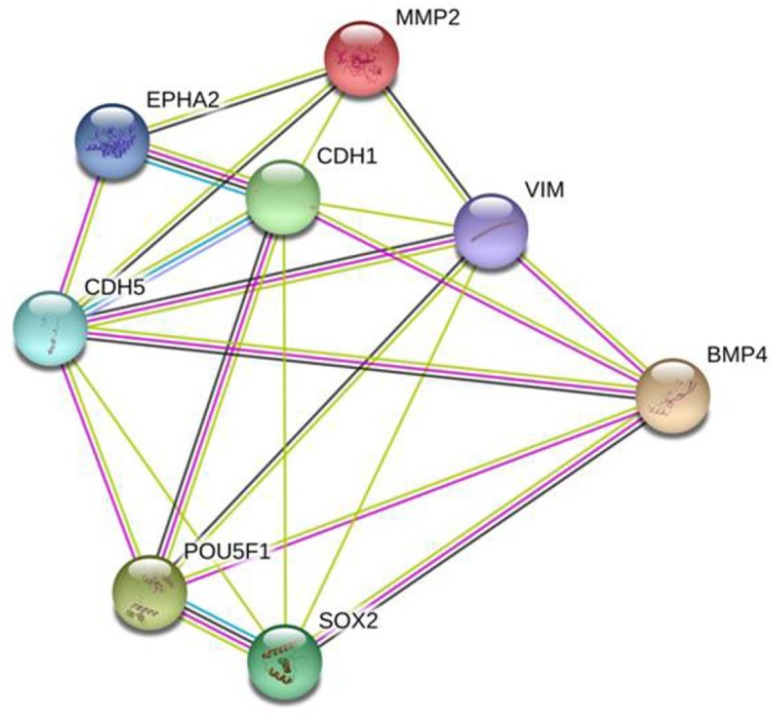
A predictive relationship between BMP4 and 7 genes related to VM formation. The network showed the interactions between BMP4, VE-cadherin (CDH5), EphA2, MMP2, E-cadherin (CDH1), vimentin (VIM), OCT4 (POU5F1) and SOX2 according to the STRING database. Nodes represent proteins. Colored lines represent the interaction between proteins. Evidence for these interactions is derived from experimental evidence (purple lines), text-mining evidence (green lines), co-expression evidence (black lines), co-occurrence evidence (navy blue lines) and databases evidence (light blue lines).

To further validate the involvement of BMP4 in VM formation, we used the STRING database to construct a PPI network that identified interactions between BMP4 and 7 genes involved in VM formation. According to the network, BMP4 directly or indirectly interacted with VE-cadherin (CDH5), EphA2, MMP2, E-cadherin (CDH1), vimentin (VIM), OCT4 (POU5F1) and SOX2 (Fig. 7). Thus, we speculate that BMP4 might promote VM formation through modulating these 7 genes.

## Discussion

This is the first study to report a connection between BMP4 and VM formation in HCC. The presence of VM signifies an alternative method for tumor cells to receive blood supply other than angiogenesis 5, 48. Previous reports have indicated that VM is related to the invasiveness and metastasis of various carcinomas 6, 49, 50. In particular, VM formation is linked to short survival in HCC 4. In our report, we observed that BMP4 was overexpressed in HCC samples and positively linked to age, histological differentiation, stage, and poor prognosis. Moreover, overexpression of BMP4 in HepG2 cells promoted migration, invasion and formation of 3D networks. We also found that down-regulation of BMP4 in SMMC7721 cells undermined the capabilities of migration, invasion and formation of VM channels. The accumulating evidence showed that BMP4 exerted an enormous influence on cell migration and invasiveness as well as the formation of VM in HCC. From HCC samples, we also observed that BMP4 expression was positively associated with VM networks. This result further demonstrated that the plasticity of some HCC cells, which are needed for VM formation, enhanced by BMP4 through a specific molecular pathway.

EMT, which loses epithelial abilities and obtains the mesenchymal properties, is known to be beneficial to invade, expand and disseminate in carcinomas. It is characterized molecularly by inhibition of E-cadherin and induction of vimentin 51-54. Previous reports have suggested that the formation of VM is positively related to the EMT in HCC. In addition, a hypothesis was put forward to describe the similarities between tumor cell plasticity in a VM network to the embryonic vasculogenesis network formation 5. Furthermore, some reports have demonstrated that the EMT possessed stem cell-like features. Thus, VM formation during EMT may also display stem-like characteristics. In the present study, overexpression of BMP4 in HepG2 cells led to EMT process as well as increased stem cell-associated protein OCT4 and SOX2. On the contrary, in SMMC7721-shBMP4 cells, EMT state was reversed and decreases in OCT4 and SOX2 expression were observed. Therefore, we concluded that BMP4 could induce VM formation by promoting EMT process and increasing stem cell-related protein expression.

EphA2, an epithelial receptor and tyrosine kinase, has been shown to be important in the VM formation. VE-cadherin, as a cell adhesion molecule, is also positively associated with VM formation 55. A lack of VE-cadherin failed to induce VM networks 56. In the present report, the expression levels of EphA2 and VE-cadherin were increased following BMP4 up-regulation and reduced following BMP4 down-regulation. Our findings were further supported by a positive relationship between BMP4 expression level and MMP2 activity, a downstream protein to EphA2/VE-cadherin signaling that acts as an important part in VM formation 57. Accumulating evidence from this report implied that EphA2 and VE-cadherin up-regulation activated MMP2 through BMP4, which then promoted cell migration, invasion, and the molding process, thereby causing VM formation. Our results have provided support to the following processes: BMP4→EphA2/VE-cadherin/MMP2→VM. This pathway may be a crucial cascade in the VM formation in HCC.

In conclusion, VM is a specialized mechanism that supports tumor proliferation and invasiveness but does not respond to available angiogenic inhibitors. Our previous studies and other similar studies have started to uncover the effect of VM formation in cancers and the VM related factors. But the exact mechanism in VM formation is still unclear. On the basis of the effect of VM on hepatocellular carcinoma, we presented in this study a potential mechanism for VM formation in HCC. We have shown that BMP4 was capable of promoting VM formation in HCC through induction of EMT stemness and modulating the EphA2/VE-cadherin/MMP2 signaling pathway. These findings were further supported by the STRING database. Consequently, BMP4 and its related molecular pathways may be worthy of further investigation and help lay the foundation for better anti-tumor therapies.

## Figures and Tables

**Table 1:** The relationship between BMP4 and VM in HCC

Variant	Total	VM		χ2	P value
	N	Negative (%)	Positive (%)		
BMP4					
Negative	40	37(92.5)	3(7.5)	5.662	0.017^*^
Positive	52	38(73.08)	14(26.92)		

P^*^<0.05

**Table 2:** The relationship between BMP4 and pathological features in HCC

Variant	Total	BMP4 expression		χ2	P value
	N	Negative (%)	Positive (%)		
Age (years)					
≤45	25	17(68.00)	8(32.00)	8.400	0.004*
>45	67	23(34.33)	44(65.67)		
Gender					
Male	80	37(46.25)	43(53.75)	1.917	0.166
Female	12	3(25.00)	9(75.00)		
Tumor size (cm)					
≤5	52	24(46.15)	28(53.85)	0.348	0.555
>5	40	16(40.00)	24(60.00)		
Histological differentiation					
I/II	31	18(58.06)	13(41.94)	4.048	0.044*
III/IV	61	22(36.07)	39(63.93)		
Stage					
I/II	38	22(57.89)	16(42.11)	5.475	0.019*
III/IV	54	18(33.33)	36(66.67)		
Metastasis					
Absent	63	28(44.44)	35(55.56)	0.076	0.783
Present	29	12(41.38)	17(58.62)		

P^*^<0.05

## Abbreviations

VM: vasculogenic mimicry
HCC: human hepatocellular carcinoma
EMT: epithelial-mesenchymal transition
MMP2: matrix metalloproteinase 2
BMPs: bone morphogenetic proteins
PBS: phosphate-buffered saline
HRP: horseradish peroxidase
PAS: periodic acid-schiff
FBS: fetal bovine serum
3D: three-dimensional
SDS-PAGE: sodium dodecyl sulfate polyacrylamide gel electrophoresis
DAPI: 4′,6-diamidino-2-phenylindole
SD: standard deviation
